# Sulphurous Acid as Applied to Wounds and Sores

**Published:** 1869-02

**Authors:** 


SULPHUROUS ACID AS APPLIED TO WOUNDS AND SORES.—Dr. Dewar, in the *Medical Times and Gazette*, recommends the application of sulphurous acid for preventing the formation of pus, and for procuring union by first intention. He sponges the wound carefully with the acid, and then dresses it with pledgets of lint saturated with the same. Prof. Syme has obtained the result claimed, by the use of sulphurous acid spray.

